# Exercise Training Promotes Cardiac Hydrogen Sulfide Biosynthesis and Mitigates Pyroptosis to Prevent High-Fat Diet-Induced Diabetic Cardiomyopathy

**DOI:** 10.3390/antiox8120638

**Published:** 2019-12-11

**Authors:** Sumit Kar, Hamid R. Shahshahan, Bryan T. Hackfort, Santosh K. Yadav, Roopali Yadav, Tyler N. Kambis, David J. Lefer, Paras K. Mishra

**Affiliations:** 1 Department of Cellular and Integrative Physiology, University of Nebraska Medical Center, Omaha, NE 68198, USA; sumit.kar@unmc.edu (S.K.); hamid.shahshahan@unmc.edu (H.R.S.); bryan.hackfort@unmc.edu (B.T.H.); santosh.yadav@unmc.edu (S.K.Y.); yadavroopali@gmail.com (R.Y.); tyler.kambis@unmc.edu (T.N.K.); 2 Department of Pharmacology and Experimental Therapeutics, Cardiovascular Center of Excellence, Louisiana State University Health Sciences Center, New Orleans, LA 70112, USA; dlefe1@lsuhsc.edu

**Keywords:** cardiac remodeling, insulin resistance, inflammasome, metabolic syndrome

## Abstract

Obesity increases the risk of developing diabetes and subsequently, diabetic cardiomyopathy (DMCM). Reduced cardioprotective antioxidant hydrogen sulfide (H_2_S) and increased inflammatory cell death via pyroptosis contribute to adverse cardiac remodeling and DMCM. Although exercise training (EX) has cardioprotective effects, it is unclear whether EX mitigates obesity-induced DMCM by increasing H₂S biosynthesis and mitigating pyroptosis in the heart. C57BL6 mice were fed a high-fat diet (HFD) while undergoing treadmill EX for 20 weeks. HFD mice developed obesity, hyperglycemia, and insulin resistance, which were reduced by EX. Left ventricle pressure-volume measurement revealed that obese mice developed reduced diastolic function with preserved ejection fraction, which was improved by EX. Cardiac dysfunction was accompanied by increased cardiac pyroptosis signaling, structural remodeling, and metabolic remodeling, indicated by accumulation of lipid droplets in the heart. Notably, EX increased cardiac H₂S concentration and expression of H₂S biosynthesis enzymes. HFD-induced obesity led to features of type 2 diabetes (T2DM), and subsequently DMCM. EX during the HFD regimen prevented the development of DMCM, possibly by promoting H₂S-mediated cardioprotection and alleviating pyroptosis. This is the first report of EX modulating H₂S and pyroptotic signaling in the heart.

## 1. Introduction

Obesity, primarily due to changes in diet to higher fat and higher sugar foods, independently increases the risk of developing heart failure [1]. Metabolic syndrome from obesity leads to type 2 diabetes (T2DM) that progresses to diastolic dysfunction and diabetic cardiomyopathy (DMCM) [2,3,4]. In rodents, however, high-fat diet (HFD) models have produced inconclusive effects on cardiac dysfunction and remodeling, and thus the molecular mechanisms by which HFD leads to DMCM are not well established [5,6,7].

Alternatively, human and rodent studies show that exercise training (EX) regimens improve cardiac function in metabolic syndrome and diabetes. EX reduces cardiac mortality in diabetic patients and increases cardiac output and contractility [8,9]. EX was found to normalize diastolic function in HFD-fed obese and T2DM mice [10,11]. Therefore, a better understanding of the molecular mechanisms induced by EX to ameliorate cardiac dysfunction would lead to novel therapeutic targets for DMCM—especially in patients unable to exercise.

Cardiomyocyte cell death is a key molecular event in the progression of DMCM, as the death of terminally differentiated cardiomyocytes leads to loss of contractile units and instigates fibrosis [12]. In metabolic syndrome, the diabetic environment of hyperglycemia, inflammatory cytokines, oxidative damage, and lipotoxicity induces several types of cell death, including non-inflammatory (apoptosis), and inflammatory (pyroptosis) cell death in the heart [13,14,15]. Pyroptosis is an inflammasome-mediated cell death mechanism where activation of the NOD-like receptor protein 3 (NLRP3) inflammasome activates caspase-1 and results in cell lysis and release of the interleukin IL-1β [16]. Hyperglycemia, fatty acids, and oxidative stress activate NLRP3 and caspase-1-mediated inflammasome, and inhibition of NLRP3 mitigates DMCM in diabetic rats [17,18,19]. Furthermore, HFD and obesity lead to inflammasome activation and infiltration of macrophages into adipose tissue, the key player in pyroptosis [18,20]. However, the role of pyroptosis in obesity-induced cardiac remodeling is unclear. Vandanmagsar et al. demonstrated that EX reduces pyroptotic cell death in adipose and liver tissue, however, this effect has not been evaluated in the heart [18].

Hydrogen sulfide (H₂S), a cardioprotective gaseous signaling molecule produced by homocysteine transsulfuration, prevents adverse cardiac remodeling and cell death, including pyroptosis [21]. H₂S inhibits caspase-1 activity and IL-1β secretion both in vitro and mouse models of inflammation [22]. It also suppresses several markers of pyroptosis in an ischemia cardiomyopathy model [23]. Notably, EX may restore bioavailability of H_2_S in hepatocytes of HFD-fed mice [24]. However, it is unknown whether H₂S is an underlying mechanism of cardioprotection via EX. 

In this study, we aimed to determine whether a HFD leads to DMCM in mice, the molecular mechanisms by which HFD leads to cardiomyopathy, and the mechanisms by which EX can mitigate HFD-induced cardiomyopathy. We tested the hypothesis that HFD-mediated obesity induces DMCM by increasing pyroptosis, and structural and metabolic remodeling, which are prevented by EX via increased biosynthesis of H₂S. 

## 2. Methods

### 2.1. Animal Models and Treatment

**Figure 1:**
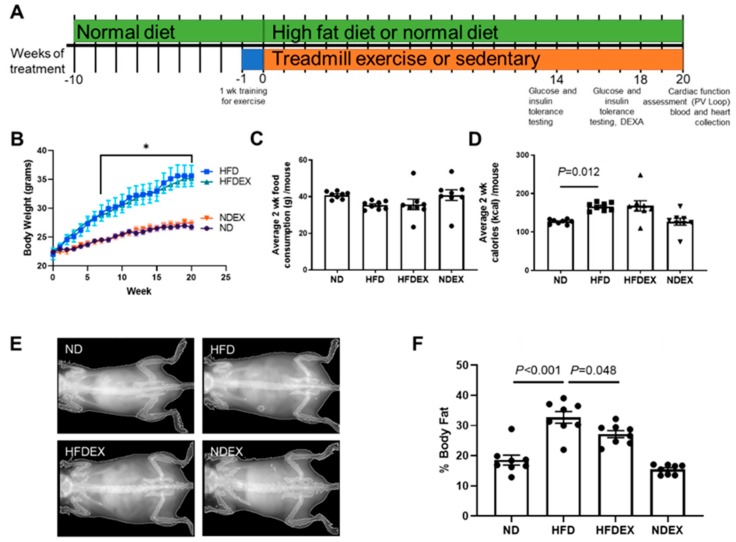
Exercise training prevents high-fat diet (HFD)-induced obesity in mice. (**A**) Timeline for diet and exercise training plan. Mice were randomly chosen for 20 weeks of high-fat diet (HFD) and exercise training (EX) at 10 weeks of age. All timepoints are reported as weeks of treatment starting at 10 weeks of age. (**B**) Average weekly body weight in the four groups of mice: normal diet (ND), HFD, HFD mice on exercise training (HFDEX), and ND mice on exercise training (NDEX). *N* = 8/group. (**C**) Food consumption over a two-week period was unchanged between groups. (**D**) Total calorie intake was increased in HFD-treated mice. (**E**) Representative DEXA imaging of mice after 18 weeks of diet and exercise training regimen. (**F**) Quantification of body fat percentage from DEXA. HFD treatment increased body fat percentage, which was prevented by exercise training. All values are expressed as mean ± SEM with dots or triangles representing each animal. One-way analysis of variance (ANOVA) and Tukey’s post-hoc test were used for statistical analysis. Repeated measures of ANOVA were used for **B**. *P < 0.05 between ND and HFD.

Ten-week-old male C57BL/6J mice (The Jackson Laboratory, Bar Harbor, Maine, USA) were randomly separated into four treatment groups: (1) normal diet (ND), (2) ND mice performing exercise training (NDEX), (3) HFD, and (4) HFD performing EX (HFDEX). The normal diet contained 17% kcal from fat and 3.1 kcal/g total (Teklad Diet 7012, Envigo, Hackensack, NJ, USA). The HFD contained 45% kcal from fat and 4.7 kcal/g total (Teklad Diet 08811, Envigo, Hackensack, NJ, USA). Mice were treated with the different diets and exercise regimens for 20 weeks (Figure 1A).

Mice were maintained in the animal facility of the University of Nebraska Medical Center with a twelve-hour light/dark cycle and provided with food and water ad-libitum. The amount of food consumed by each cage was measured on a bi-weekly basis and divided by the number of animals in the cage to estimate food intake for each mouse. Body weight of each mouse was measured weekly. 

All animal studies were performed following the guidelines of the National Institutes of Health and the protocols were approved by the Institutional Animal Care and Use Committee (IACUC # 19-079-09-FC) of the University of Nebraska Medical Center.

### 2.2. Exercise Training (EX) Regimen

Mice in EX groups underwent moderate-intensity treadmill exercise and were pre-trained for four days before the experiment regimen. The speed and duration of the treadmill run was gradually increased to adapt to the final speed with zero degrees incline (Supplementary Table S1). Mice ran on the treadmill five days/week for twenty weeks, except on the days when glucose or insulin tolerance tests were conducted. The sedentary mice were also brought to the treadmill with the EX mice, but they were kept away from the running belt. Therefore, the sedentary mice experienced all the stress of handling, noise, vibrations, and lack of food and water during the exercise training period that EX mice experienced, except for the actual running on the treadmill.

### 2.3. Dual Energy X-ray Absorbance (DEXA)

Dual energy X-ray absorptiometry (DEXA, Lunar Piximus, GE Lunar Corp, Madison, WI, USA) was performed during week 18 to measure differences in body composition. Prior to scanning, calibration was performed using the calibration phantom provided by the supplier. Mice were anesthetized with isoflurane during scanning. The DEXA system uses a cone beam X-ray source generating energies of 35 and 80 keV, and a flat detector having individual pixel dimension of 0.18 × 0.18 mm.

### 2.4. Glucose and Insulin Tolerance Tests (GTT, ITT)

We performed intraperitoneal glucose tolerance tests (GTT) after 14 and 18 weeks of treatment. Mice were fasted for seven hours prior to an intraperitoneal glucose injection (2 g glucose/kg body weight in a 20% solution in saline). Blood glucose was measured before the injection and at different time intervals (15, 30, 45, 60, 90, and 120 min) following injection by an Accu-Chek Aviva glucometer (Roche, Basel, Switzerland).

The intraperitoneal insulin tolerance test (ITT) was assessed at the same time points on different days than the GTT. An intraperitoneal injection of 0.75 U insulin/kg body weight (Humulin R, Lilly, Indianapolis, IN, USA) was given to the mice fasted for seven hours. Blood glucose levels were measured before and after insulin injection at the time intervals of 15, 30, 45, 60, and 90 min.

### 2.5. Left Ventricle Hemodynamics

Pressure-volume (PV) loop recordings were performed in the left ventricle at the end of the 20-week diet and exercise training regimen using an open chest procedure, as described previously [25]. A 4 mm 1.2 F catheter probe (Transonic) was inserted into the left ventricle via the heart apex and volume measurements were calibrated automatically by admittance from the probe. Measurements were made following the best practice guidelines for invasive hemodynamic measurement in mice [26]. Heart rate was maintained at 450 to 500 beats per minute (bpm) by adjusting anesthesia (1–2% isoflurane) and maintaining body temperature at 37 ℃. Baseline load-dependent pressure-volume recordings were made after confirming the placement of the catheter in the left ventricle. Hemodynamic parameters of diastolic and systolic function were derived by averaging at least 20 cardiac cycles from the recordings using the LabChart Pro PV Loop Analysis Add-On (ADInstruments, Colorado Springs, CO, USA).

### 2.6. Western Blotting

The left ventricle of mouse hearts previously frozen at –80 °C were homogenized with a glass homogenizer using Radioimmunoprecipitation assay buffer (RIPA, BP-115, Boston BioProducts, Ashland, MA, USA) with protease and phosphatase inhibitor (MSSAFE, Sigma, St. Louis, MO, USA). 25–30 ug of protein were loaded onto sodium dodecyl sulfate–polyacrylamide gel electrophoresis (SDS PAGE) gels. Membranes were blocked with 5% milk for at least 30 min, incubated with primary antibody overnight at 4 °C and secondary antibody for 2 h at room temperature. Band intensity was determined using Bio-Rad Image Lab software. Protein expression was normalized against total protein measured by Ponceau staining, as cardiac remodeling may alter housekeeping protein expression [27].

The primary antibodies used were: IL-1β (1:1000, 52718S, Cell Signaling, Danvers, MA, USA), Caspase-1 (1:1000, ab1872, Abcam, Cambridge, MA, USA), NLRP3 (1:1000, NBP2-12446, Novus, Littleton, CO, USA), apoptosis-associated speck-like protein (ASC, 1:100, sc-54414, Santa Cruz, Dallas, TX, USA), β-Myosin Heavy Chain (β-MHC, 1:1000, ab172967, Abcam), cystathionine-gamma-lyase (CSE, 1:1500, H00001491-M02, Abnova, Taipei, Taiwan), Cystathionine-β-synthase (CBS, 1:1000, H00000875-M01, Abnova), Homocysteine (1:1000, ab15154, Abcam), 3-mercaptopyruvate sulfurtransferase (MPST, 1:100, ab85377, Abcam, ), SP-1 (1:1000, 07-645, Millipore, St. Louis, MO, USA), phospho-SP-1 (1:1000, ab59257, Abcam), BiP (1:1000, 3177, Cell Signaling), CHOP (1:1000, ab141019, Abcam), GLUT4 (1:2500, ab654, Abcam) and TGF-β1 (1:100, sc146, Santa Cruz, CA, USA). The secondary antibodies used were anti-mouse IgG-HRP, and anti-rabbit IgG-HRP (used at half the concentration of the respective primary antibody dilution, Cell Signaling). 

### 2.7. Gene Expression Assays

RNA was isolated from the left ventricle using the mirVana isolation kit (AM1560, Thermo Fisher, Waltham, MA, USA) for both total and small RNA. For mRNA quantification, cDNA was synthesized with iScript cDNA synthesis (Bio-Rad, Hercules, CA, USA) and amplified using SYBR® green in a CFX® qPCR instrument (Bio-Rad, Hercules, CA, USA). We used 18s rRNA as an endogenous control. The primer sequences were: 18s (NR_003278), forward 5′GTAGTTCCGACCATAAACGA3′ and reverse 5′TCAATCTGTCAATCCTGTCC3′, and CBS (NM_144855) forward 5′TGCGGAACTACATGTCCAAG3′ and reverse- 5′TTGCAGACTTCGTCTGATGG3′.

cDNA for miRNA was synthesized using the TaqMan^®^ microRNA reverse transcription kit (Life Technologies, USA, catalog # 4366597). cDNA was amplified with TaqMan primers for miR-133a, miR-29b, and miR-30a and the endogenous control U6 snRNA (Thermo Fisher). Gene expression was quantified using CFX Manager 3.0 software (Bio-Rad).

### 2.8. Electron Microscopy and Histology

5 µm transverse paraffin heart sections were used for picrosirius red staining, which stains collagen in red. Staining was performed by the University of Nebraska Medical Center Tissue Sciences Core Facility. A VWR (Radnor, PA, USA) bright-field microscope was used for imaging slides. The same area of the left ventricle was imaged for each heart. Transmission electron microscopy (TEM) of sections of the left ventricle fixed with EM grade Trump’s fixative was performed with a Tecnai G2 Spirit® transmission electron microscope (FEI Company, Hillsboro, OR, USA).

### 2.9. Hydrogen Sulfide Measurement

H₂S was measured in serum and tissue using gas chromatography, as previously described [28]. Previously frozen blood or homogenized tissue was incubated with 1 M sodium citrate buffer at 37 °C with shaking to release H₂S before gas chromatography analysis. Concentrations of H₂S were calculated against a standard curve of Na_2_S, and tissue concentrations were normalized against mg of protein added to the buffer using bicinchoninic acid assay (BCA) protein measurement. 

### 2.10. Statistical Analyses

To compare the means from four groups, we used one-way analysis of variance (ANOVA), followed by Tukey’s post-hoc test, using GraphPad Prism 8.0 (La Jolla, CA, USA). Repeated measures of ANOVA were used when the same measurements were taken multiple times on an animal over time. Samples and data analysis were blinded or performed by different analysts wherever possible. Sample sizes (8 animals/group) were determined by power calculation prior to commencement of the study based on variability of historical data.

## 3. Results

### 3.1. High-Fat Diet Induces Obesity and Type 2 Diabetes (T2DM) Phenotype in Mice

To validate HFD-induced obesity in mice, body weight and body fat were monitored during the treatment regimens. Mice started at the same average body weight across all groups. HFD caused a rapid increase in body weight within eight weeks when compared to the mice on a normal diet (ND) (Figure 1B). At the end of the 20-week diet regimen, HFD-fed mice gained an average of 15.6 g from the start of the regimen when compared to ND-fed mice, which only gained an average of 4.33 g. This corresponded to the increased body fat percent in HFD mice when measured with DEXA (Figure 1E,F). After 18 weeks of HFD feeding, mice had an average body fat percentage of 32.7% ± 1.94% when compared to their ND-fed littermates, which had 18.5% ± 1.65% body fat. DEXA imaging demonstrated that the increase in body fat occurred primarily in the abdominal area, confirming visceral obesity.

Exercise training during HFD feeding slightly reduced body weight in HFD mice, which was not statistically significant. However, exercise training reduced body fat percentage in HFD mice (20.4% reduction). Average grams of food consumed over two weeks was the same across all groups of mice (Figure 1C), suggesting that weight gain was due to the increased fat content of the diet itself and not increased consumption.

**Figure 2:**
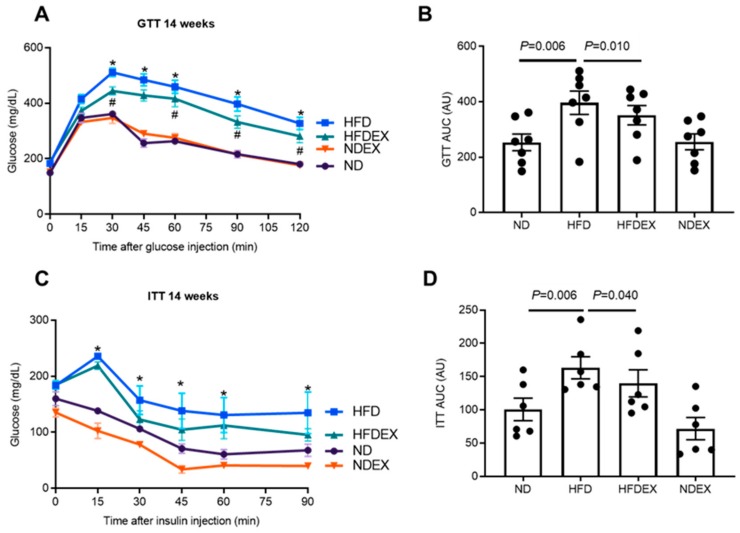
Exercise training prevents HFD-induced development of T2DM phenotype. (**A**) and (**B**) Intraperitoneal glucose tolerance test (GTT) at 14-week treatment in the four groups of mice: normal diet (ND), high-fat diet (HFD), HFD mice on exercise training (HFDEX), and ND mice on exercise training (NDEX). Blood glucose was measured for 2 h at several time points after the injection of glucose. Glucose clearance was decreased in HFD but improved with EX. *N* = 8. (**C**) and (**D**) Intraperitoneal insulin tolerance test (ITT) at 14-week treatment. Blood glucose concentrations were measured for 1.5 h after the injection of insulin. Insulin resistance was higher in HFD as compared to the ND group. * *P* < 0.05 between ND and HFD. # *p* < 0.05 between HFDEX and HFD. All values are expressed as mean ± SEM with dots representing each animal. One-way ANOVA and Tukey’s post-hoc test were used for statistical analysis.

The HFD-induced obesity in mice led to features of T2DM. Fasting baseline blood glucose was elevated in HFD mice (Table 1). Impaired glucose tolerance was observed in obese mice after 14 weeks of the diet regimen during glucose tolerance tests (GTT). Plasma glucose concentrations were elevated from 30 min to 120 min after glucose injection in HFD mice when compared to ND mice (Figure 2A, B). Insulin sensitivity was also impaired with HFD at 14 weeks, as HFD mice demonstrated elevated glucose for 90 min after the injection of insulin (Figure 2 C, D). Glucose intolerance remains similar after 18 weeks of the HFD regimen (Supplementary Figure S1). Exercise led to improvements in glucose handling. Fasting glucose was reduced in HFD mice with exercise, glucose tolerance was improved at 14 weeks, and insulin sensitivity showed a trend toward improvement (Figure 2A–D).

### 3.2. Exercise Prevents High-Fat Diet (HFD)-Induced Cardiac Dysfunction in Mice

**Figure 3:**
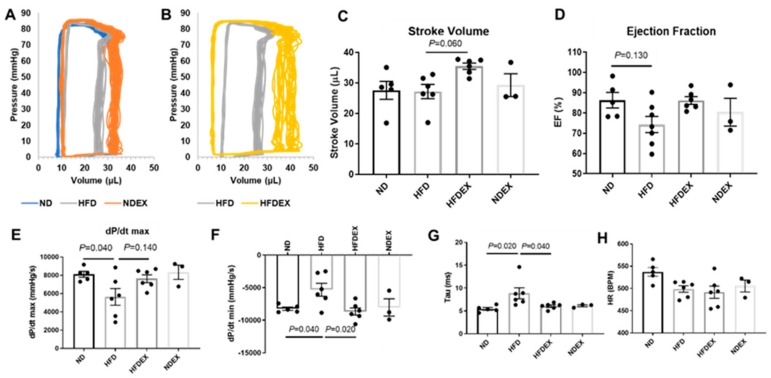
Exercise training improves diastolic left ventricle dysfunction in diet- induced obese mice. (**A**) Representative PV trace in ND, HFD, and NDEX mice. (**B**) Representative PV trace in HFD mice (trace replicated from **A**) and HFDEX mice. (**C**) Stroke volume was elevated in HFDEX mice compared to HFD. (**D**) Ejection fraction (EF) was not changed with HFD or EX. (**E**) Maximum change in pressure over time (dP/dt_max_) was reduced with HFD. (**F** and **G**) Measures of diastolic function (dP/dt_min_ and Tau) were worsened with HFD but normalized in HFDEX mice. (**H**) Heart rate (HR) during the recordings was constant between groups. All values are expressed as mean ± SEM with dots representing each animal. One-way ANOVA and Tukey’s post-hoc test were used for statistical analysis.

Systolic and diastolic function of the left ventricle was measured using PV loop recordings after 20 weeks of diet and exercise regimens (Figure 3). HFD-induced obesity led to an impairment of diastolic function when compared to ND-fed mice. The change in left ventricular pressure over time (dP/dt_min_), a measure of relaxation, was elevated in HFD mice when compared to ND mice (Figure 3F), delineating a lower rate of relaxation. The exponential decay of the ventricular pressure during isovolumic relaxation (Tau) was lengthened in obese mice (Figure 3G), also indicating impaired diastolic function. dP/dt_max_, a measure of systolic contractility, decreased with HFD. Ejection fraction was unchanged in all groups, suggesting that cardiac dysfunction had not yet manifested into systolic heart failure. Heart rate during hemodynamics measurement was unchanged between groups (Figure 3H).

Exercise training in obese mice prevented the deterioration of ventricular function. It increased stroke volume only in HFD-fed mice (comparison of HFDEX with HFD) (Figure 3C) and also normalized dP/dt_min_ and Tau (Figure 3G).

### 3.3. Obesity-Induced Structural and Metabolic Cardiac Remodeling

**Figure 4:**
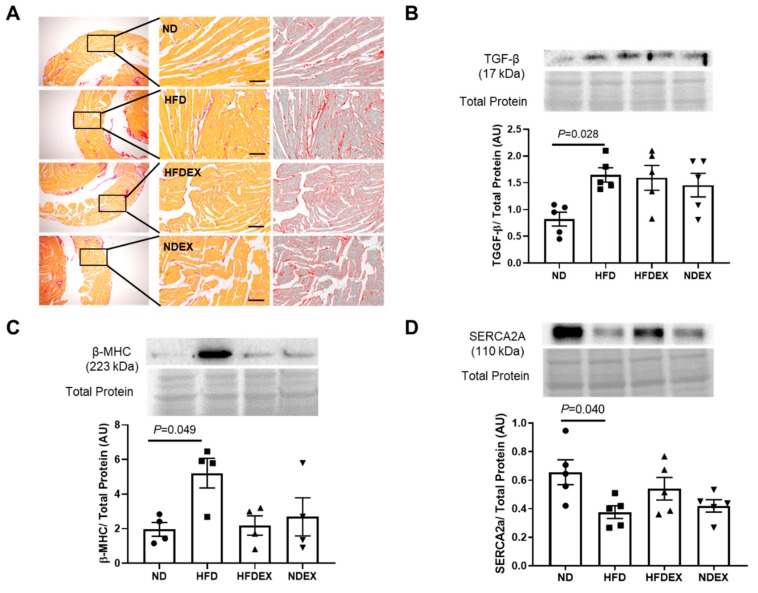
High- fat diet upregulates markers of cardiac structural remodeling. (**A**) Representative sirius red staining of a section of the left ventricle for interstitial collagen fibers was unaltered in all the four groups. Scale bar = 200 µm. A similar portion of the left ventricular wall was imaged in all groups. (**B**) Western blot quantification of the pro-fibrotic signaling marker TGF-β protein expression in left ventricle tissue demonstrates an upregulation in HFD mice compared to ND. (**C**) Western blot quantification of the hypertrophy marker β-MHC protein expression in left ventricle tissue demonstrates an upregulation in HFD mice compared to ND. (**D**) Western blot quantification of contractile protein sarco/endoplasmic reticulum Ca^2+^-ATPase (SERCA-2A) protein expression in left ventricle tissue demonstrates a downregulation in HFD mice compared to ND. All values are expressed as mean ± SEM with dots of different shape representing each animal in a group. One-way ANOVA and Tukey’s post-hoc test were used for statistical analysis.

To determine whether HFD-induced impairment in diastolic function was due to the remodeling of the heart, we evaluated structural, metabolic, and molecular remodeling events. To determine structural and molecular remodeling, we measured cardiac fibrosis and hypertrophy and their molecular markers. Picrosirius red staining was performed on sections of the left ventricle to stain for collagen fibers. No change in interstitial fibrosis was observed among the four groups (Figure 4A). However, pro-fibrotic signaling molecule transforming growth factor beta-1 (TGFβ) was elevated in the heart of the HFD-fed obese mice (Figure 4B). Also, the pathological hypertrophy marker β-myosin heavy chain (β-MHC) was elevated in the left ventricle of the obese mice (Figure 4C). However, the gross weight of the hearts normalized to their respective body weights and tibia lengths were unchanged between groups (Table 1). Exercise training in obese mice did not significantly improve these structural remodeling pathways (Figure 4). It did not prevent the HFD-induced increase in the expression of β-MHC (Figure 4C) and the reduction in the expression of sarco/endoplasmic reticulum Ca^2+^-ATPase (SERCA2A) (Figure 4D), which is involved in cardiomyocytes calcium handling and cardiac contractility.

**Figure 5:**
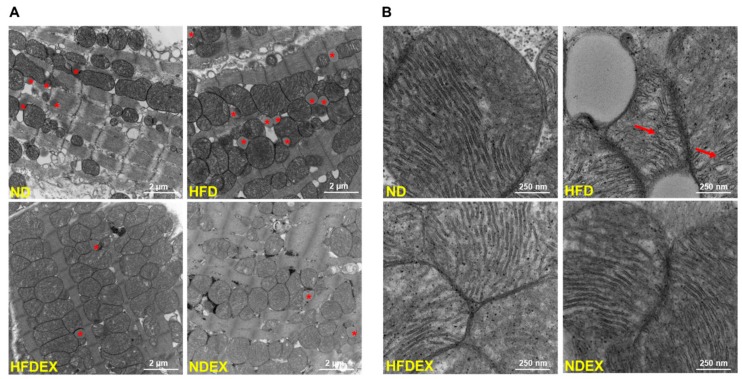
Exercise training prevents high- fat diet- induced metabolic remodeling. (**A**) Representative electron micrographs in a section of left ventricle tissue. * shows a lipid droplet which were increased in quantity and size in the HFD heart section but reduced with exercise training. (**B**) Higher magnification focusing on mitochondria in the left ventricle. Mitochondrial cristae structure is deformed in HFD compared to ND, as indicated by arrows. *N* = 2/group.

Further, metabolic remodeling was observed using EM sections of the left ventricle (Figure 5). HFD obese mice had a greater number of lipid droplets (ND versus HFD, indicated by a star) (Figure 5A), which were also larger in size compared to normal diet-fed mice, presenting signs of lipotoxicity. These lipid droplets were located adjacent to mitochondria. Furthermore, HFD obese mice had an irregular and swollen mitochondrial structure with deformed cristae (ND versus HFD, indicated by an arrow) (Figure 5B). However, cardiac protein expression of fatty acid metabolism regulators peroxisome proliferator-activated receptors (PPAR), PPARα, and PPARγ were unaltered between groups (Supplementary Figure S2). Exercise training reduced the number of lipid droplets and improved mitochondrial structure (Figure 5A, B).

### 3.4. Exercise Prevents HFD-Induced Cardiac Pyroptosis

**Figure 6:**
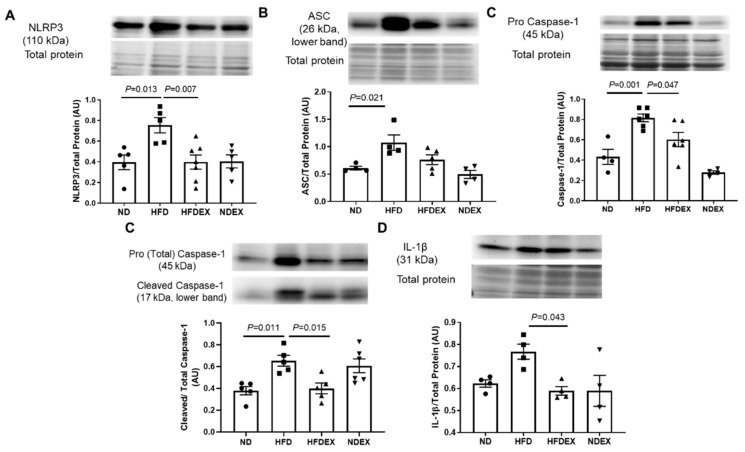
Exercise training prevents HFD-induced upregulation of cardiac pyroptosis. Western blotting was performed to determine the components of inflammasome complex and pyroptosis in the left ventricle of mice hearts. (**A**) HFD-fed obese mice show upregulation of the NLRP3 inflammasome in the left ventricle, which is prevented by exercise training. (**B**) Apoptosis-associated speck adaptor protein (ASC) that assembles with NLRP3 and caspase-1 to make inflammasome complex is elevated in the heart of obese mice but attenuated by exercise training. (**C**) Exercise training prevents HFD-induced upregulation of cardiac caspase-1. (**D**) Expression of IL-1β, which is upregulated by HFD, is normalized by exercise training. All values are expressed as mean ± SEM with dots of different shape representing each animal in a group. One-way ANOVA and Tukey’s post-hoc test were used for statistical analysis.

Pyroptosis precedes cardiac remodeling and dysfunction and is induced by lipotoxicity and mitochondrial damage. In the left ventricle of the four groups of mice, we evaluated the following components of pyroptosis: inflammasome formation (NLRP3), caspase activation (caspase-1 cleavage), and elevation of inflammatory cytokine IL-1β, which is activated by caspase-1 during pyroptosis. Inflammasome formation and activation were observed in the HFD group by elevation in protein expression of NLRP3 and apoptosis-associated speck adaptor protein (ASC) (Figure 6A,B). Expression of pro-caspase-1 and the ratio of activated caspase-1 to total caspase-1 were also elevated in obese mice (Figure 6C), indicating the activation of pyroptosis. Finally, a pro-inflammatory response in the heart due to pyroptosis was observed by an increase in the expression of IL-1β (Figure 6D). Exercise training prevented HFD-induced upregulation of NLRP3, ASC pro-caspase-1 and IL-1β, and activation of caspase-1 (Figure 6A–D). These results demonstrate that obesity-induced cardiac inflammasome formation, pyroptosis activation, and pro-inflammatory response, all of which were prevented by an exercise training regimen.

### 3.5. Exercise Training Induces Biosynthesis of Cardioprotective Hydrogen Sulfide

We evaluated two signaling pathways to determine potential mechanisms for the prevention of pyroptosis and remodeling by EX in obese mice: endoplasmic reticulum (ER) stress responses and H₂S signaling. Binding immunoglobulin protein (BiP), which mediates ER response to unfolded proteins and promotes cell survival, was unchanged in expression in obese mice (Supplementary Figure S3A). However, exercise training induced expression of BiP in obese mice. On the other hand, expression of the transcription factor CHOP, which induces cell death during ER stress, was unchanged in all four groups (Supplementary Figure S3B). 

**Figure 7:**
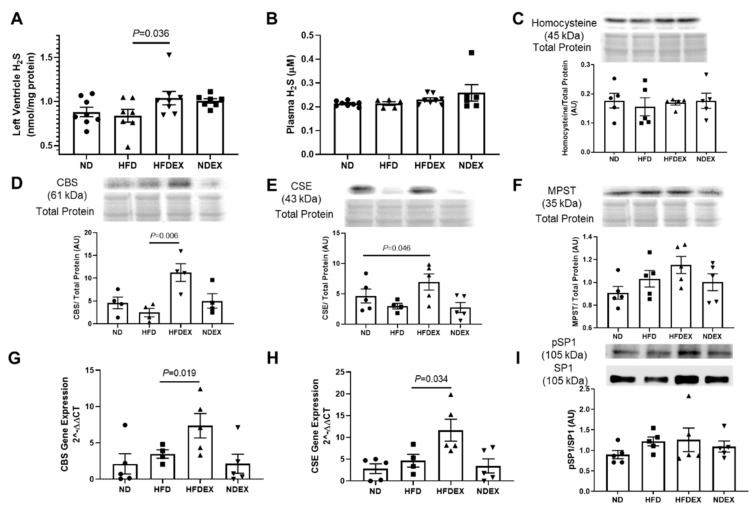
Exercise training increases biosynthesis of hydrogen sulfide in the heart of obese mice. Exercise training increased the concentration of H₂S in mice fed with both a normal and high-fat diet in the left ventricle tissue of the heart (**A**) but not in plasma (**B**). Homocysteine, the precursor substrate for H₂S production, was unaltered among the four groups (**C**). Protein expression of the H₂S-producing transulfuration enzyme CBS was increased in the left ventricle of HFDEX mice compared to HFD mice (**D**). Protein expression of another H₂S-producing transulfuration enzyme, CSE, was also increased in the left ventricle of HFDEX mice compared to HFD mice (**E**). The final H₂S-producing enzyme, MPST, localized in mitochondria was unchanged between groups (**F**). Transcriptional activation of H_2_S-producing enzymes, CBS and CSE, was observed with increased mRNA expression in HFDEX (**G, H**), however activation of the transcriptional factor SP1 that activates CSE was unchanged (**I**). All values are expressed as mean ± SEM with dots of different shape representing each animal in a group. One-way ANOVA and Tukey’s post-hoc test were used for statistical analysis.

H₂S concentrations were elevated in heart tissue of obese mice undergoing exercise training (Figure 7A). Obesity itself did not alter H₂S concentrations compared to ND-fed mice (Figure 7A). The biosynthesis pathway for H₂S generation was also modulated by exercise training. Cardiac levels of homocysteine, which is metabolized to produce H₂S, was unchanged in all four groups (Figure 7C). However, two of the enzymes which produce H₂S from homocysteine by the transulfuration pathway were elevated in the hearts of HFDEX mice: cystathionine-β-synthase (CBS) and cystathionine-gamma-lyase (CSE) (Figure 7D,E). A third H₂S-generating enzyme localized in mitochondria, 3-mercaptopyruvate sulfur-transferase (MPST), did not show changes in expression with exercise training (Figure 7F). The transcript levels of CBS and CSE genes were also increased by exercise training in HFD-fed mice (Figure 7G,H). Finally, SP-1, a transcription factor which binds to the CSE promoter, was marginally elevated in HFDEX mice, but was not statistically significant (Figure 7I). HFD itself did not change H₂S biosynthesis enzymes, and H₂S biosynthesis enzymes were also unaltered in ND mice undergoing exercise (Figure 7C–I).

## 4. Discussion

**Figure 8:**
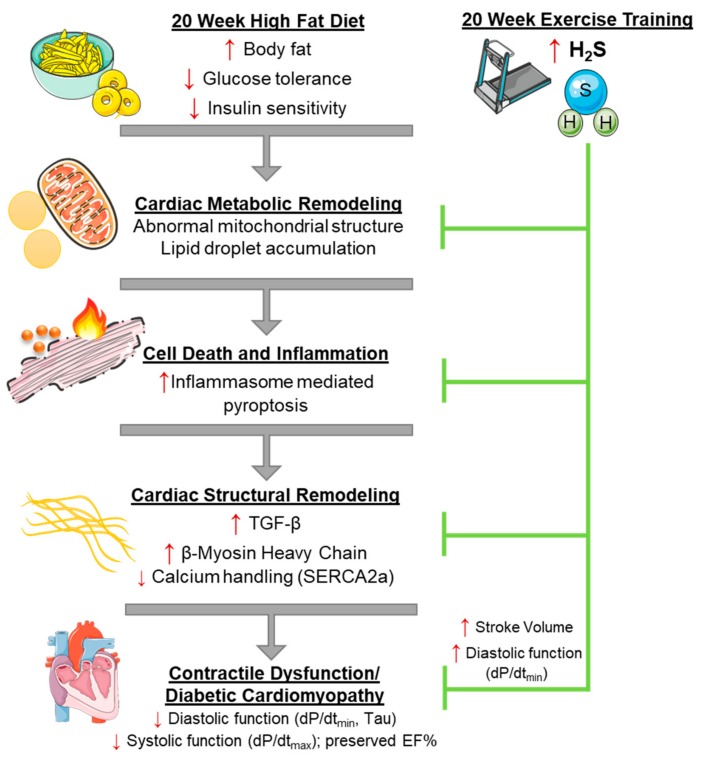
Working model for the amelioration of cardiac dysfunction by exercise training in high-fat diet-fed obese mice. High-fat diet increases obesity and develops the type 2 diabetes (T2DM) phenotype that promotes cardiac metabolic remodeling, resulting in activation of inflammasome-mediated pyroptosis, a cell death mechanism. This leads to adverse cardiac remodeling signaling, including structural changes such as fibrosis formation and hypertrophy of cardiomyocytes that follow to fill the growing vacant extracellular space, and functional impairment due to reduced calcium handling through SERCA-2A that leads to cardiac dysfunction. Exercise training prevents HFD-induced development of the T2DM phenotype and upregulation of cardiac pyroptosis, as well as adverse cardiac remodeling, possibly by increasing cardioprotective H₂S signaling.

In this study, we demonstrated that a 20-week high-fat diet treatment to C57BL6 mice led to obesity and a T2DM phenotype with insulin resistance. This diet treatment induced systolic and diastolic dysfunction of the left ventricle while maintaining ejection fraction, similar to the heart failure with preserved ejection fraction condition. Further analysis demonstrated that cardiac dysfunction was due in part to pro-fibrotic and hypertrophic signaling, damaged mitochondrial structure, lipid droplet accumulation, and an increase in pyroptosis. Importantly, a moderate intensity EX training regimen prevented cardiac dysfunction, metabolic remodeling, and pyroptosis activation. An increase in H₂S synthesis in the myocardium may explain the cardioprotection of EX in the heart. These findings are summarized in Figure 8.

Our obese mice demonstrated a majority of the criteria for DMCM, as described by the Animal Models of Diabetic Complications Consortium [29]. This included elevated fasting glucose (213 ± 13.5 mg/dL in HFD mice), insulin resistance (GTT and ITT), diastolic dysfunction (dp/dt min measured by catheterization), molecular evidence of fibrosis and hypertrophy (increased TGF-β and decreased SERCA-2A and β-MHC), and evidence of lipotoxicity. Other diet-induced DMCM models with HFD or high sucrose diets have produced mixed phenotypes [6]. HFD in rodent models seems to consistently deteriorate diastolic and systolic function measured after 6 to 16 weeks of treatment [30,31]. Some diet-induced models show marked hypertrophy of the heart [30], while others only observe elevated levels of molecular markers of hypertrophy and fibrosis with no histological signs, similar to our study [32,33]. Variations in the severity of DMCM in diet-induced models may be due to differences in the caloric and fat contents of diets, the duration of treatment, and the varied genetic background of the mice.

Identification and targeting of non-apoptotic forms of cell death in DMCM is crucial, since estimates show only 80 cardiomyocytes out of 10^5^ undergo apoptosis in a failing heart [34,35]. Activation of pyroptosis in obesity and metabolic syndrome is well-established as the release of IL-1β accelerates pancreatic β cell death [36]. In our study, HFD activated the three main components of pyroptotic cell death: inflammasome formation, caspase-1 activation, and inflammation by the production of IL-1β. In addition, mitochondrial damage and lipid droplet accumulation, as we observed in the left ventricle, are potent activators of the cardiac inflammasome and pyroptosis [37]. Elevation of serum lipids results in lipotoxicity or cardiac steatosis in the form of lipid droplets, which accumulate near mitochondria [38]. The uptake of lipids is stimulated by activation of PPARγ, and accumulated lipids are vulnerable to the formation of lipid peroxides and ceramides, which damage mitochondrial proteins and activate the inflammasome [18,39,40]. We demonstrated that exercise training reduces lipotoxicity and pyroptosis in the HFD-fed mice heart. While our data suggests this reduction from exercise training was not mediated by altering PPARγ signaling, it may prevent HFD-induced cardiac pyroptosis by promoting cardioprotective signaling of H₂S.

H₂S donor supplementation has been shown to reduce pyroptosis activation in the retina, bone marrow, and heart under different pathological conditions [22,41,42]. Our study complements a study by Wang et al., which demonstrated that exercise training increased H₂S in the liver by upregulating enzymes involved in H₂S biosynthesis, particularly by stimulating the transulfuration pathway [24]. Similarly, increased cardiac H₂S in our study were accompanied by increased cardiac expression of H₂S biosynthetic enzymes. Changes in H₂S were not due to changes in homocysteine, a non-protein coding amino acid which is a precursor of H₂S and can be altered by diet [43]. Several mechanisms have been demonstrated for how H₂S protects against cell death, including by the reduction of inflammatory cytokines such as IL-1β [44], stimulating antioxidant production [45], and downregulating autophagy [46].

Endoplasmic reticulum (ER) stress may also play a role in obesity-induced DMCM and the cardioprotective mechanisms of exercise. Ceramide accumulation from lipotoxicity has been shown to induce protein misfolding and ER stress, which is an activator of the inflammasome and IL-1β release in T2DM [47,48]. ER stress responses may also mediate the cardioprotective mechanisms of exercise training and H₂S. A human study showed that exercise training reduced activation of different arms of the unfolded protein response in obesity [49]. Another study demonstrated that H₂S supplementation in HFD-fed mice reduced ER stress in the heart [50]. However, in our study, the effect of HFD and exercise training on ER stress was inconclusive. CCAAT-enhancer-binding protein homologous protein (CHOP), a marker of chronic ER stress, was not elevated in HFD mice. HFDEX mice showed a marked increase in expression of BiP/GrP78, a chaperone which senses and initiates adaptive unfolded protein responses. It is possible that other ER stress pathways are elevated or the lower percentage of dietary fat (45%) did not induce as severe ER stress as in other studies, which have used 60% fat [50]. Nevertheless, the role of ER stress in linking exercise training and H₂S signaling in DMCM merits further investigation.

This study demonstrates that a high-fat diet induces obesity and DMCM in mice. In this diet-induced model of DMCM, cardiac dysfunction was accompanied by abnormal mitochondrial structure, lipotoxicity, and activation of pyroptosis. Targeting and inhibiting pyroptosis could be a therapeutic option for DMCM. Importantly, exercise training reduced pyroptosis signaling and concurrently enhanced H₂S biosynthesis. H₂S donor treatment to the heart failure patients has demonstrated encouraging results in the phase I clinical trial [51]. Thus, inducing H₂S signaling pathways could benefit those patients with DMCM who are unable to exercise. Further clinical evidence is required to extrapolate the evidence of this study on the benefits of H₂S on patients with DMCM.

## Figures and Tables

**Table 1:** Gravimetric characteristics of mice with 20 weeks of normal and high-fat diet with or without exercise training.

Parameter	ND (*n* = 8)	HFD (*n* = 8)	HFDEX (*n* = 8)	NDEX (*n* = 8)
Body Weight (g)	26.7 ± 0.57	35.6 ± 2.13 *	35.2 ± 1.04 *	27.2 ± 0.43
Heart Weight (mg)	124 ± 5.92	137 ± 6.82	146 ± 7.01	125 ± 3.43
HW/TL (mg/mm)	6.87 ± 0.35	7.81 ± 0.31	8.23 ± 0.38	7.08 ± 0.18
Fasting Glucose (mg/dL)	162 ± 9.13	213 ± 13.5 *	182 ± 13.5 #	136 ± 10.4

Values are expressed as mean ± SEM. ND = normal diet, HFD = high-fat diet, HFDEX = high-fat diet with exercise training, NDEX= normal diet with exercise training, HW = heart weight, TL = tibia length. * *P* < 0.05 versus ND. # *P* < 0.05 versus HFD.

## Supplementary Materials

The following are available online at https://www.mdpi.com/2076-3921/8/12/638/s1, Figure S1: 18 week treatment of high fat diet induces a diabetic phenotype in mice, Figure S2: Exercise did not alter PPAR lipid metabolism regulator expression, Figure S3: Expression of endoplasmic reticulum stress markers in obese mice with exercise, Table S1: Exercise training protocol: Regimen for the exercise pre-training and training.
