# Supplementary material for: Exercise Training Promotes Cardiac Hydrogen Sulfide Biosynthesis and Mitigates Pyroptosis to Prevent High-Fat Diet-Induced Diabetic Cardiomyopathy
Source: Antioxidants (Basel). 2019 Dec 11;8(12):638. doi: 10.3390/antiox8120638 (PMC6943713; doi:10.3390/antiox8120638)
Supplement: Supplementary file 1 [file antioxidants-08-00638-s001.pdf]

**Supplementary File**

# Exercise promotes cardiac hydrogen sulfide biosynthesis and mitigates pyroptosis to prevent high-fat diet induced diabetic cardiomyopathy

Sumit Kar[1]*, Hamid R. Shahshahan[1*], Bryan T. Hackfort[1*], Santosh K. Yadav[1], Roopali Yadav[1], Tyler N. Kambis[1], David J. Lefer[2], Paras K. Mishra[1]

Affiliations:

[1]Department of Cellular and Integrative Physiology, University of Nebraska Medical Center, Omaha, NE, USA

[2]Cardiovascular Center of Excellence, Louisiana State University Health Sciences Center, New Orleans, LA 70112

**Corresponding author:**

Paras Kumar Mishra, Ph.D., F.A.H.A. F.C.V.S.
Department of Cellular and Integrative Physiology
University of Nebraska Medical Center
Omaha, NE-68198, USA
Phone: 402-559-8524
Fax: 402-559-4438
Email: paraskumar.mishra@unmc.edu

*Authors contributed equally

**Table S1.** Exercise training protocol: Regimen for the exercise pre-training and training.

| **Exercise** | **Warm-up** | | **Run** | | **Cool-down** | |
|---|---|---|---|---|---|---|
| regimen | Time (min) | Speed (M/min) | Time (min) | Speed (M/min) | Time (min) | Speed (M/min) |
| Pre-training | 5 | 8 | 5, 20, | 10, 10 | 5 | 8 |
| 1-4 days | | | 30, 40 | 12, 12 | 5 | 8 |
| Week 1 | 5 | 8 | 50 | 12 | 5 | 8 |
| Week 2-20 | 5 | 8 | 50 | 15 | 5 | 8 |

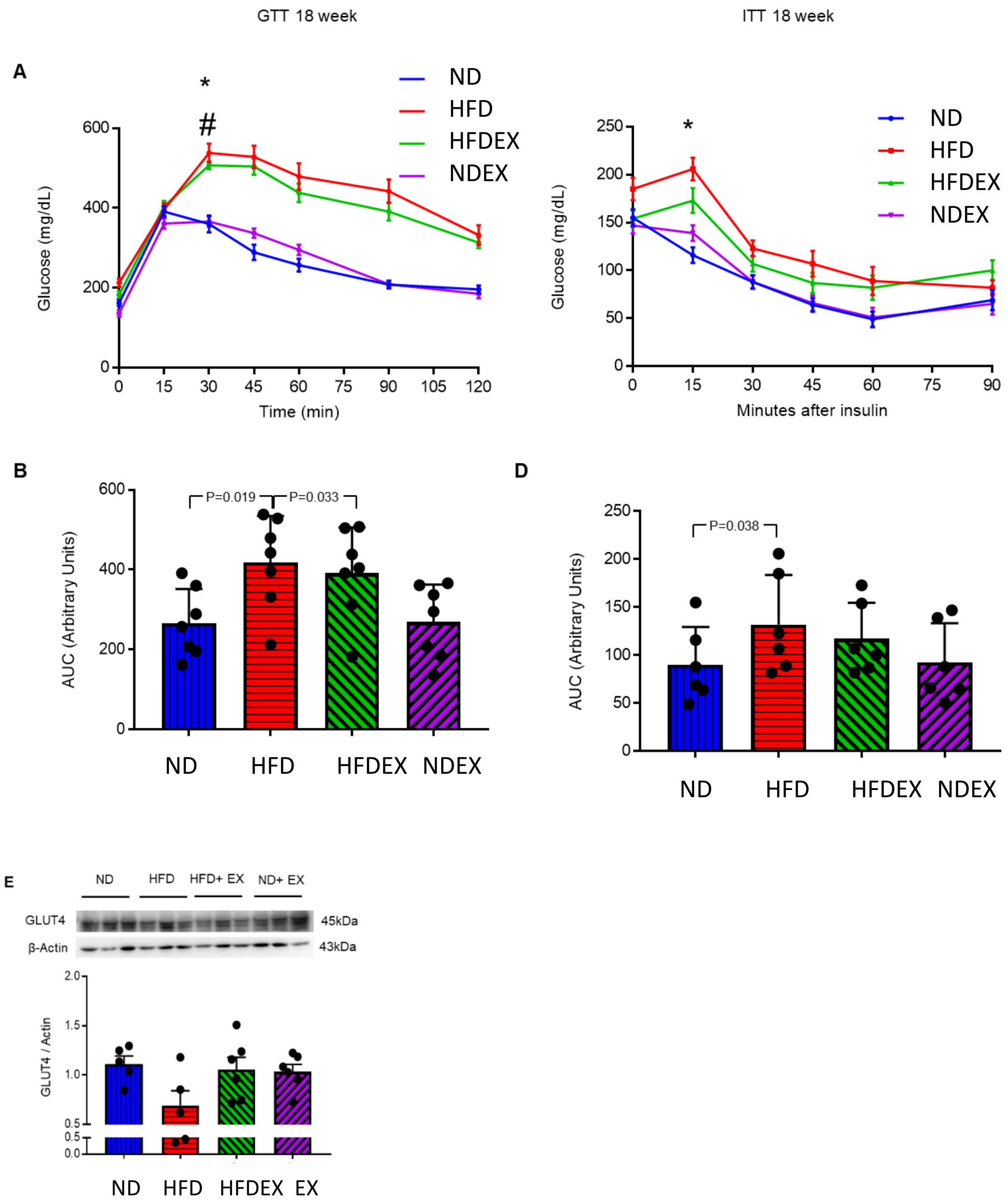


**Figure S1. 18 weeks treatment of high-fat diet induces a diabetic phenotype in mice. A, B.** Intraperitoneal glucose tolerance test (GTT) at 18-week treatment in the four groups of mice: normal diet (ND), high-fat diet (HFD), HFD mice on exercise training (HFDEX) and ND mice on exercise training (NDEX). Glucose clearance was decreased in HFD but improved with EX. **C, D.** Intraperitoneal insulin tolerance test (ITT) at 18-week treatment. Insulin resistance was higher in HFD as compared to ND group which was also improved with EX. **E.** Glucose transporter type 4 (GLUT-4) expression in the heart was slight reduced with HFD treatment but not statistically significant. *, $P<0.05$ between ND and HFD. #, $P<0.05$ between HFDEX and HFD. All values expressed as mean±SEM with dots representing each animal. Repeated measures one-way ANOVA and Tukey's post-hoc test was used for statistical analysis.

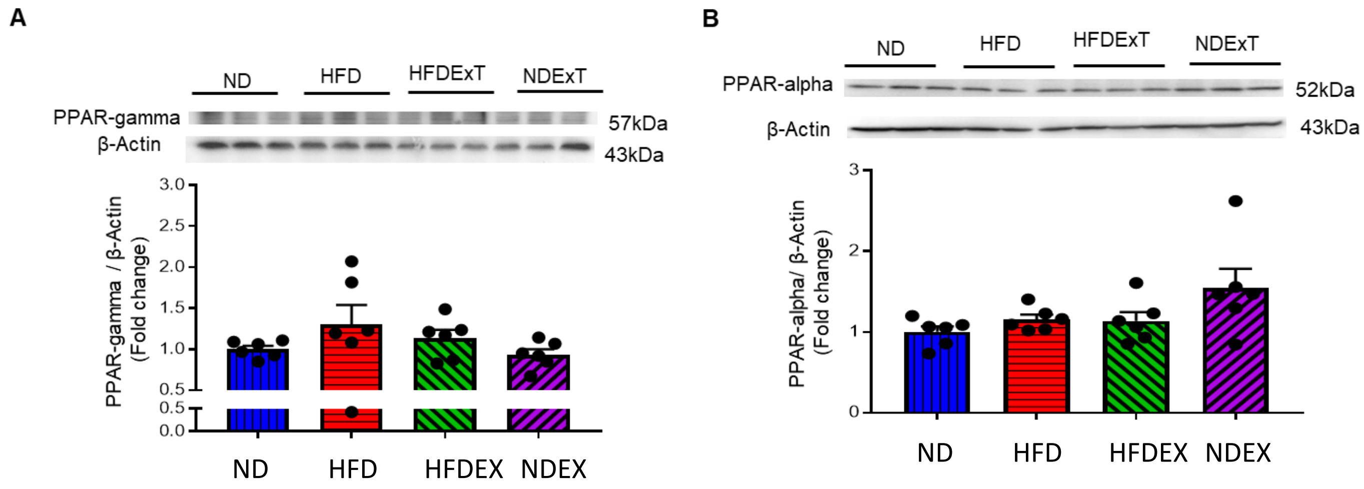


**Figure S2. Exercise training did not alter lipid metabolism regulator PPAR expression.** PPARα and PPARγ expression were unaltered by HFD or EX. All values expressed as mean±SEM with dots representing each animal. Repeated measures one-way ANOVA and Tukey's post-hoc test was used for statistical analysis.

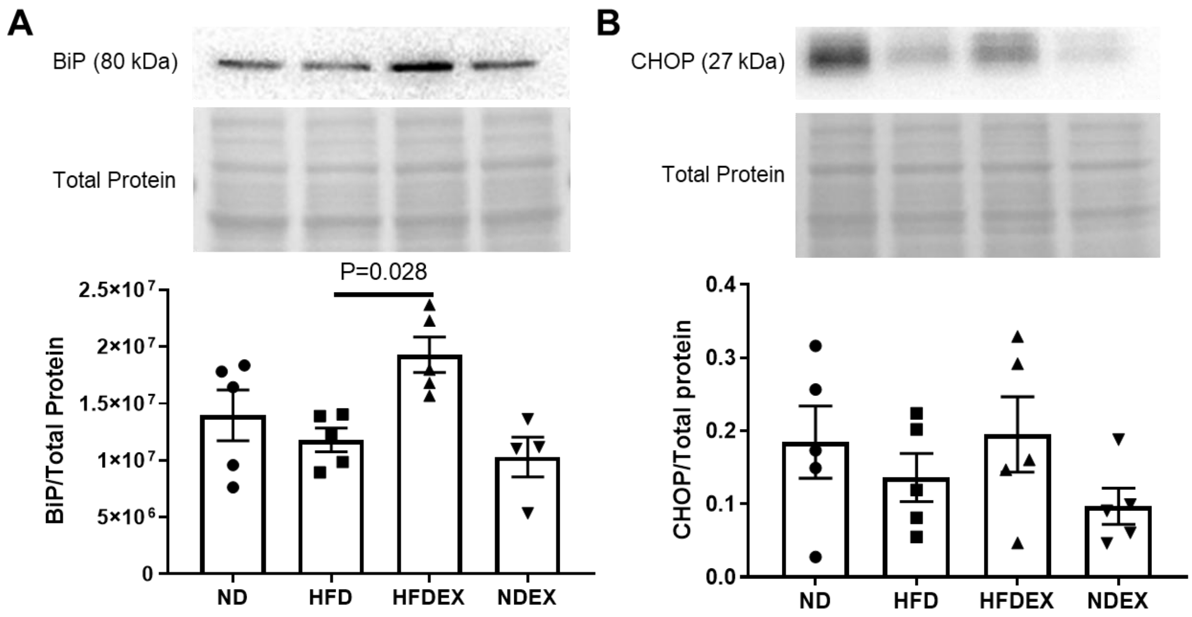


**Figure S3. Expression of endoplasmic reticulum stress markers BiP and CHOP in obese mice with exercise training.** All values expressed as mean±SEM with dots representing each animal. One-way ANOVA and Tukey's post-hoc test was used for statistical analysis.
